# Estimating sparse regression models in multi-task learning and transfer learning through adaptive penalisation

**DOI:** 10.1093/bioinformatics/btaf406

**Published:** 2025-07-17

**Authors:** Armin Rauschenberger, Petr V Nazarov, Enrico Glaab

**Affiliations:** Bioinformatics and Artificial Intelligence, Department of Medical Informatics, Luxembourg Institute of Health (LIH), 1445 Strassen, Luxembourg; Biomedical Data Science, Luxembourg Centre for Systems Biomedicine (LCSB), University of Luxembourg, 4362 Esch-sur-Alzette, Luxembourg; Bioinformatics and Artificial Intelligence, Department of Medical Informatics, Luxembourg Institute of Health (LIH), 1445 Strassen, Luxembourg; Multiomics Data Science, Department of Cancer Research, Luxembourg Institute of Health (LIH), 1445 Strassen, Luxembourg; Biomedical Data Science, Luxembourg Centre for Systems Biomedicine (LCSB), University of Luxembourg, 4362 Esch-sur-Alzette, Luxembourg

## Abstract

**Method:**

Here, we propose a simple two-stage procedure for sharing information between related high-dimensional prediction or classification problems. In both stages, we perform sparse regression separately for each problem. While this is done without prior information in the first stage, we use the coefficients from the first stage as prior information for the second stage. Specifically, we designed feature-specific and sign-specific adaptive weights to share information on feature selection, effect directions, and effect sizes between different problems.

**Results:**

The proposed approach is applicable to multi-task learning as well as transfer learning. It provides sparse models (i.e. with few non-zero coefficients for each problem) that are easy to interpret. We show by simulation and application that it tends to select fewer features while achieving a similar predictive performance as compared to available methods.

**Availability and implementation:**

An implementation is available in the R package “sparselink” (https://github.com/rauschenberger/sparselink, https://cran.r-project.org/package=sparselink).

## 1 Background

Here, we are concerned with related high-dimensional regression problems for numerical prediction or binary classification. In high-dimensional settings, where the number of features is much larger than the sample size, it can be difficult to estimate accurate predictive models. If it is not possible to increase the sample size available for model training, it might be beneficial to share information among related problems to increase the predictive performance. In this work, we use the term *multi-task learning* to mean “solving related problems about the same features and the same samples” and the term *transfer learning* to mean “solving related problems about the same features but different samples.”

To illustrate the difference between these definitions of multi-task and transfer learning, we consider two sets, each consisting of two related regression problems. For simplicity, we assume the targets are numerical and centred (i.e. linear regression with zero intercept). Each target is then modelled as a linear combination of the features plus an error term. In the case of multi-task learning, we have


$$\left(\begin{array}{cc} y_{1,1} & y_{1,2}\\ \vdots & \vdots \\ y_{n,1} & y_{n,2} \end{array}\right)=\left(\begin{array}{ccc} x_{1,1} & \cdots & x_{1,p}\\ \vdots & & \vdots \\ x_{n,1} & \cdots & x_{n,p} \end{array}\right)\times \left(\begin{array}{cc} \beta_{1,1} & \beta_{1,2}\\ \vdots & \vdots \\ \beta_{p,1} & \beta_{p,2} \end{array}\right)+\left(\begin{array}{cc} \epsilon_{1,1} & \epsilon_{1,2}\\ \vdots & \vdots \\ \epsilon_{n,1} & \epsilon_{n,2} \end{array}\right)\;,$$

where both problems concern the same *n* samples and the same *p* features. While each problem has its own vector for the target ($y_{\cdot ,1}$ or $y_{\cdot ,2}$), the unknown slopes ($\beta_{\cdot ,1}$ or $\beta_{\cdot ,2}$), and the unknown errors ($\epsilon_{\cdot ,1}$ or $\epsilon_{\cdot ,2}$), both models have a common feature matrix (X). By contrast, in the case of transfer learning, we have


$$\begin{array}{c} \begin{array}{ccccccccc} & \left(\begin{array}{c} y_{1}^{\left(1\right)}\\ \vdots \\ y_{n}^{\left(1\right)} \end{array}\right) & = & \left(\begin{array}{ccc} x_{1,1}^{\left(1\right)} & \cdots & x_{1,p}^{\left(1\right)}\\ \vdots & & \vdots \\ x_{n,1}^{\left(1\right)} & \cdots & x_{n,p}^{\left(1\right)} \end{array}\right) & \times & \left(\begin{array}{c} \beta_{1,1}\\ \vdots \\ \beta_{p,1} \end{array}\right) & + & \left(\begin{array}{c} \epsilon_{1,1}^{\left(1\right)}\\ \vdots \\ \epsilon_{n,1}^{\left(1\right)} \end{array}\right) & \\ \text{and} & \left(\begin{array}{c} y_{1}^{\left(2\right)}\\ \vdots \\ y_{m}^{\left(2\right)} \end{array}\right) & = & \left(\begin{array}{ccc} x_{1,1}^{\left(2\right)} & \cdots & x_{1,p}^{\left(2\right)}\\ \vdots & & \vdots \\ x_{m,1}^{\left(2\right)} & \cdots & x_{m,p}^{\left(2\right)} \end{array}\right) & \times & \left(\begin{array}{c} \beta_{1,2}\\ \vdots \\ \beta_{p,2} \end{array}\right) & + & \left(\begin{array}{c} \epsilon_{1,2}^{\left(2\right)}\\ \vdots \\ \epsilon_{m,2}^{\left(2\right)} \end{array}\right) & , \end{array} \end{array}$$

where the two problems concern the same *p* features but different *n* or *m* samples, respectively. Each problem has not only its own vector for the target ($y^{\left(1\right)}$ or $y^{\left(2\right)}$), the unknown slopes ($\beta_{\cdot ,1}$ or $\beta_{\cdot ,2}$), and the unknown errors ($\epsilon^{\left(1\right)}$ or $\epsilon^{\left(2\right)}$), but also its own feature matrix ($X^{\left(1\right)}$ or $X^{\left(2\right)}$). For both multi-task and transfer learning, however, we expect that the unknown slopes ($\beta_{\cdot ,1}$ and $\beta_{\cdot ,2}$) are positively correlated, provided the problems are sufficiently related. When fitting these models, it can therefore be beneficial to share information between the two problems (e.g. by shrinking each pair of slopes towards a common value).

We previously proposed methods for multi-task learning (Rauschenberger and Glaab 2021) and transfer learning (Rauschenberger *et al.* 2023) that increase the predictive performance of penalised regression by sharing information between related prediction or classification problems. Here we are also concerned with multi-task and transfer learning, but our focus is on sparse models. We want to estimate models with few non-zero coefficients, which are easy to interpret. For example, if a method selects 10 out of 20,000 genes for predicting an outcome, interpreting the estimated effects of the genes on the outcome is straightforward. A researcher can then examine the signs and sizes of the estimated effects for all selected genes one by one.

Here, we propose a simple two-stage procedure for estimating sparse models in high-dimensional multi-task or transfer learning settings. In both stages, we perform penalised regression separately for each problem. While this is done with standard elastic net regularisation (Zou and Hastie 2005) in the first stage, we use the initial coefficients from the first stage to construct feature-specific and sign-specific penalty factors for the second stage. These penalty factors depend on the same problem (internal weights), as for the adaptive lasso (Zou 2006), and on the other problems (external weights), as for the weighted lasso (Bergersen *et al.* 2011). If the problems are sufficiently related, this information exchange should increase the predictive performance of the models while maintaining their sparsity.

## 2 Methods

### 2.1 Notation

Suppose there are *q* datasets, indexed by k∈{1,…,q}, with the same set of *p* features, indexed by j∈{1,…,p}. In each dataset *k*, the samples are indexed by $i\in \{1,\ldots ,n_{k}\}$. We assume that all datasets include the same samples for multi-task learning (i.e. the total sample size is $n=n_{1}=\cdots =n_{q}$) and that each dataset includes different samples for transfer learning (i.e. the total sample size is $n=\sum_{k=1}^{q}n_{k}$). Let $y_{i}^{\left(k\right)}$ be the value of the target for sample *i* in dataset *k*, and let $x_{i,j}^{\left(k\right)}$ be the value of feature *j* for sample *i* in dataset *k*. For each dataset, this leads to an $n_{k}\times 1$ target vector $y^{\left(k\right)}$, with one entry for each sample, and an $n_{k}\times p$ feature matrix $X^{\left(k\right)}$, with samples in the rows and features in the columns. In the case of multi-task learning, we could use a more compact notation with a common n×q outcome matrix, namely $Y=(y^{\left(1\right)}\|\ldots \|y^{\left(q\right)})$, and a common n×p feature matrix, namely $X=X^{\left(1\right)}=\cdots =X^{\left(q\right)}$.

### 2.2 Model

For each dataset *k*, the aim is to fit the generalised linear model


$$h\left(E\left[y_{i}^{\left(k\right)}|x_{i,\cdot }^{\left(k\right)}\right]\right)=\beta_{0,k}+\sum_{j=1}^{p}\beta_{j,k}x_{i,j}^{\left(k\right)}\;,$$

where *h* denotes a link function (e.g. identity for Gaussian family or logit for binomial family), $E[y_{i}^{\left(k\right)}|x_{i,\cdot }^{\left(k\right)}]$ denotes the expected value of $y_{i}^{\left(k\right)}$ conditional on $x_{i,\cdot }^{\left(k\right)}$, $\beta_{0,k}$ denotes the unknown intercept, and $\beta_{j,k}$ denotes the unknown slope of feature *j*. In low-dimensional settings ($p\ll n_{k}$), we could estimate the unknown parameters (intercept and slopes) by maximising the family-dependent likelihood $L(y^{\left(k\right)};\beta_{\cdot ,k})$, but high-dimensional settings ($p\gg n_{k}$) require penalisation.

### 2.3 Stage 1 - Estimation without prior information

In the first stage, we estimate the unknown parameters without sharing information between different problems. This involves penalising the likelihood with the sum of the absolute values ($L_{1}$-norm, lasso) and the sum of the squared values ($L_{2}$-norm, ridge) of the slopes:


$$\rho_{\text{init}}(\lambda_{1,k};\beta_{\cdot ,k})=\lambda_{1,k}\;\sum_{j=1}^{p}\left[\alpha \left|\beta_{j,k}\right|+(1-\alpha )\frac{\beta_{j,k}^{2}}{2}\right]\;,\;\;$$

where the hyperparameter $\lambda_{1,k}$ is restricted to be non-negative ($\lambda_{1,k}\geq 0$) and determines the shrinkage, with $\lambda_{1,k}=0$ leading to an unpenalised model and $\lambda_{1,k}=\infty$ leading to an intercept-only model (i.e. ${\hat{\beta }}_{1,k}=\cdots ={\hat{\beta }}_{p,k}=0$). We set the elastic net mixing parameter not to α=1 (lasso) but to α=0.95 (lasso-like elastic net) to allow the model to select more features than there are samples and to select strongly correlated features. If lasso-like elastic net regularisation sets all coefficients equal to zero—such as under small sample sizes or many weak effects—ridge regularisation (α=0) might be a better choice. We estimate the parameters by optimising the penalised likelihood $L(y^{\left(k\right)};\beta_{\cdot ,k})-\rho_{\text{init}}^{\left(k\right)}(\lambda_{1,k};\beta_{\cdot ,k})$ with respect to the vector $\beta_{\cdot ,k}$. Let ${\hat{\beta }}_{0,k}$ denote the estimated intercept in problem *k*, and let ${\hat{\beta }}_{j,k}$ denote the estimated slope for feature *j* in problem *k*.

### 2.4 Prior information

We use the coefficients from the initial regressions to construct penalty factors for the final regressions. For each problem *k*, we consider the initial coefficients from the same problem (*k*) and those from the other problems (l≠k) separately. As an initial positive coefficient is evidence for a positive effect and evidence against a negative effect, and the other way round for initial negative coefficients, we derive different weights for positive effects and negative effects in the final regressions. For the internal information (i.e. obtained from the same problem), the idea is that the initial coefficient determines the weight for a final coefficient of the same sign. Let $w_{j,k}^{\text{int}}$ and $w_{p+j,k}^{\text{int}}$ indicate the internal weights for a final positive or negative coefficient, respectively, for feature *j* in problem *k*. For the external information (i.e. obtained from the other problems), the idea is that all positive and all negative initial coefficients determine the weight for a final positive or negative coefficient, respectively. Let $w_{j,k}^{\text{ext}}$ and $w_{p+j,k}^{\text{ext}}$ indicate the weights for the final positive or negative coefficient, respectively, for feature *j* in problem *k*. The internal and external weights (columns) for estimating a positive or a negative effect (rows) are


$$\begin{array}{ccc} & \;\text{internal}\; & \;\text{external}\;\\ + & \;w_{j,k}^{\text{int}}=\max(0,{\hat{\beta }}_{j,k}) & \;w_{j,k}^{\text{ext}}=\sum_{l\neq k}\max(0,{\hat{\beta }}_{j,l})\;\\ - & \;w_{p+j,k}^{\text{int}}=|\min(0,{\hat{\beta }}_{j,k})|\; & \;w_{p+j,k}^{\text{ext}}=|\sum_{l\neq k}\min(0,{\hat{\beta }}_{j,l})|\; \end{array}\;\;.$$

By construction, for feature *j* in problem *k*, only one internal weight can be non-zero ($I[w_{j,k}^{\text{int}}\neq 0]+I[w_{p+j,k}^{\text{int}}\neq 0]\in \{0,1\}$) but both external weights can be non-zero ($I[w_{j,k}^{\text{ext}}\neq 0]+I[w_{p+j,k}^{\text{ext}}\neq 0]\in \{0,1,2\}$). Specifically, if a feature obtains a positive or negative initial coefficient in the same problem, one of its internal weights is non-zero, and if a feature obtains at least one positive and at least one negative initial coefficient in the other problems, both external weights are non-zero.

### 2.5 Stage 2 - Estimation with prior information

In the second stage, we re-estimate the unknown parameters (intercepts and slopes) while sharing information between problems. We do this by decomposing each slope into a positive and a negative part ($\beta_{j,k}=\gamma_{j,k}-\gamma_{p+j,k}$), where both parts are constrained to be non-negative ($\gamma_{j,k}\geq 0$ and $\gamma_{p+j,k}\geq 0$), and by penalising each part differentially.

The model is represented by


$$h\left(E\left[y_{i}^{\left(k\right)}|x_{i,\cdot }^{\left(k\right)}\right]\right)=\gamma_{0,k}+\sum_{j=1}^{p}\left(\gamma_{j,k}-\gamma_{p+j,k}\right)x_{i,j}^{\left(k\right)}\;,$$

with the non-negativity constraint


$$\gamma_{j,k}\geq 0\;,\;\forall j\in \{1,\ldots ,2p\}\;,$$

where $\gamma_{j,k}$ and $\gamma_{p+j,k}$ denote the effects of feature *j*. Again, estimating the family-dependent likelihood $L(y^{\left(k\right)};\gamma_{\cdot ,k})$ requires penalisation.

We penalise the likelihood with a weighted sum of the non-negative slopes:


$$\rho_{\text{final}}(\lambda_{2,k},\delta_{k}^{\text{int}},\delta_{k}^{\text{ext}};\gamma_{\cdot ,k})=\lambda_{2,k}\sum_{j=1}^{2p}\frac{\gamma_{j,k}}{{\left(w_{j,k}^{\text{int}}\right)}^{\delta_{k}^{\text{int}}}+{\left(w_{j,k}^{\text{ext}}\right)}^{\delta_{k}^{\text{ext}}}}\;,$$

where the hyperparameter $\lambda_{2,k}(\geq 0)$ determines the total amount of shrinkage, and the hyperparameters $\delta_{k}^{\text{int}}(\geq 0)$ and $\delta_{k}^{\text{ext}}(\geq 0)$ determine the scaling of the internal and the external weights, respectively, as in the adaptive lasso (Zou 2006) and the weighted lasso (Bergersen *et al.* 2011), respectively. As $\lambda_{2,k}$ increases, more and more coefficients are set to zero, until reaching an intercept-only model ($\gamma_{1,k}=\cdots =\gamma_{2p,k}=0$). The numerator ${\left(w_{j,k}^{\text{int}}\right)}^{\delta_{k}^{\text{int}}}+{\left(w_{j,k}^{\text{ext}}\right)}^{\delta_{k}^{\text{ext}}}$ represents the prior weight of feature *j* in problem *k*. If the exponent $\delta_{k}^{\text{int}}$ or $\delta_{k}^{\text{ext}}$ equals zero, the internal or external weights, respectively, have no impact on the prior weights, as any base raised to the power of zero equals one. The model can thereby ignore the internal weights from the same problem or the external weights from the other problems. Exponents less than one dampen the influence of large internal or external weights more than that of smaller ones.

**Figure 1.**
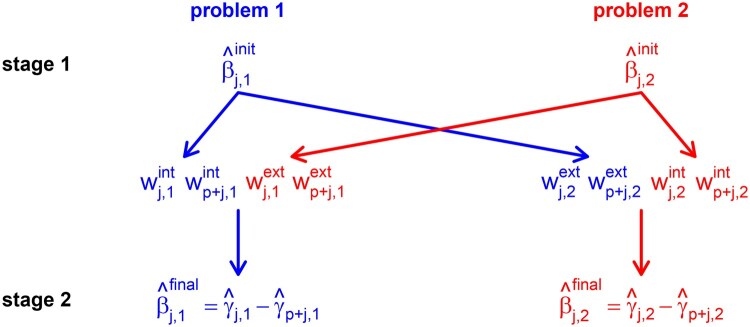
Information flow. The initial coefficients from the first-stage regressions (top) determine the internal and external weights for the second-stage regressions (centre) and thereby influence the final coefficients from the second-stage regressions (bottom). This example is for two problems (blue, red). If there are three (or more) problems, the external weights are based on the other two (or more) problems. In each problem *k*, each feature *j* obtains two internal and two external weights, namely for a positive effect (indexed by *j*) and for a negative effect (indexed by p+j).

We estimate the parameters by optimising the penalised likelihood $L(y^{\left(k\right)};\gamma_{\cdot ,k})-\rho_{\text{init}}^{\left(k\right)}(\lambda_{2,k};\gamma_{\cdot ,k})$ with respect to the vector $\gamma_{\cdot ,k}$. For problem *k*, the final estimated intercept is ${\hat{\gamma }}_{0,k}$ and the final estimated slope for feature *j* is ${\hat{\gamma }}_{j,k}-{\hat{\gamma }}_{p+j,k}$. As ${\hat{\gamma }}_{j,k}(\geq 0)$ and ${\hat{\gamma }}_{p+j,k}(\geq 0)$ are estimated for the same feature (perfect multicollinearity) under penalisation, at least one of ${\hat{\gamma }}_{j,k}$ and ${\hat{\gamma }}_{p+j,k}$ will equal zero ($I[{\hat{\gamma }}_{j,k}\neq 0]+I[{\hat{\gamma }}_{p+j,k}\neq 0]\leq 1$). Figure 1 shows the flow of information from the first stage to the second stage.

### 2.6 Hyperparameter optimisation

The proposed approach includes the hyperparameters $\lambda_{1,k}$ and $\lambda_{2,k}$ for determining the shrinkage in the first and the second stage, respectively, and the hyperparameters $\delta_{k}^{\text{int}}$ and $\delta_{k}^{\text{ext}}$ for determining the scaling of the internal and external weights, respectively. We use sequences of up to 100 candidate values for the regularisation parameters $\lambda_{1,k}$ and $\lambda_{2,k}$ (Friedman *et al.* 2010, R package glmnet) and the candidate values {0.0,0.2,0.4,0.6,0.8,1.0} for the scaling parameters $\delta_{k}^{\text{int}}$ and $\delta_{k}^{\text{ext}}$. We use grid search with 10-fold internal cross-validation for optimising these four hyperparameters, for each problem *k*, although other approaches (e.g. random search) or other numbers of folds (e.g. leave-one-out cross-validation) are also possible. While cross-validation involves excluding a fold for all problems (targets) in the case of multi-task learning, it involves excluding a fold for one problem (dataset) in the case of transfer learning. This is because multi-task learning involves multiple problems for the same samples, but transfer learning involves multiple problems for different samples. Hyperparameter optimisation is relatively fast due to cyclical coordinate descent along the regularisation path (Friedman *et al.* 2010, R package glmnet). Table 1 includes the pseudocode for (hyper)parameter optimisation.

**Table 1.** Pseudocode for (hyper)parameter optimisation in the proposed approach to multi-task learning (left) and transfer learning (right).^a^

Multi-task learning	Transfer learning
**Require:**	**Require:**
n×q matrix Y (targets)	$n_{k}\times 1$ vectors $y^{\left(k\right)}$ (targets), ∀k∈{1,…,q}
n×p matrix X (features)	$n_{k}\times p$ matrices $X^{\left(k\right)}$ (features), ∀k∈{1,…,q}
**Ensure:**	**Ensure:**
q×1 vectors $\lambda_{1}$, $\lambda_{2}$, $\delta^{\text{int}}$, $\delta^{\text{ext}}$ (hyperparameters)	q×1 vectors $\lambda_{1}$, $\lambda_{2}$, $\delta^{\text{int}}$, $\delta^{\text{ext}}$ (hyperparameters)
(p+1)×q matrix $\hat{B}$ (coefficients)	(p+1)×q matrix $\hat{B}$ (coefficients)
set n×1 vector of fold identifiers fold,	set $n_{k}\times 1$ vector of fold identifiers fold${\;}_{k}$ ,
with values from 1 to 10	with values from 1 to 10, ∀k∈{1,…,q}
initialise various empty n×q matrices $\hat{Y}$	initialise various empty $n_{k}\times 1$ vectors ${\hat{y}}^{\left(k\right)}$, ∀k∈{1,…,q}
**Hyperparameter optimisation**	**Hyperparameter optimisation**
**for** *k* in 1 to *q* **do**	**for** *k* in 1 to *q* **do**
$\lambda_{1,k}\leftarrow \mathrm{ElasticNetCV}(Y_{\cdot ,k},X,\mathrm{fold})$	$\lambda_{1,k}\leftarrow \mathrm{ElasticNetCV}(y^{\left(k\right)},X^{\left(k\right)},\mathrm{fol}d_{k})$
**end for**	**end for**
**for** *i* in 1 to 10 **do**	**for** *k* in 1 to *q* **do**
**for** *k* in 1 to *q* **do**	${\hat{B}}_{\cdot ,k}\leftarrow \mathrm{ElasticNet}(y^{\left(k\right)},X^{\left(k\right)},\lambda_{1,k})$
${\hat{B}}_{\cdot ,k}^{-\kappa (i)}\leftarrow \mathrm{ElasticNet}(Y_{\cdot ,k}^{-\kappa (i)},X^{-\kappa (i)},\lambda_{1,k})$	**end for**
**end for**	**for** *i* in 1 to 10 **do**
**for** *k* in 1 to *q* **do**	**for** *k* in 1 to *q* **do**
$\left\{w_{k}^{\text{int}},w_{k}^{\text{ext}}\}\leftarrow \mathrm{Weights}({\hat{B}}^{-\kappa (i)},k\right)$	${\hat{B}}^{-\kappa (i)}\leftarrow \hat{B}$
**for** various $\lambda_{2,k}$, $\delta_{k}^{\text{int}}$, $\delta_{k}^{\text{ext}}$ **do**	${\hat{B}}_{\cdot ,k}^{-\kappa (i)}\leftarrow \mathrm{ElasticNet}(y^{\left(k),-\kappa (i\right)},X^{\left(k),-\kappa (i\right)},\lambda_{1,k})$
$z_{k}\leftarrow \mathrm{PenaltyFactors}(w_{k}^{\text{int}},w_{k}^{\text{ext}},\delta_{k}^{\text{int}},\delta_{k}^{\text{ext}})$	$\left\{w_{k}^{\text{int}},w_{k}^{\text{ext}}\}\leftarrow \mathrm{Weights}({\hat{B}}^{-\kappa (i)},k\right)$
${\hat{\Gamma }}_{\cdot ,k}^{-\kappa (i)}\leftarrow \mathrm{AdCoLasso}(Y_{\cdot ,k}^{-\kappa (i)},X^{-\kappa (i)},\lambda_{2,k},z_{k})$	**for** various $\lambda_{2,k}$, $\delta_{k}^{\text{int}}$, $\delta_{k}^{\text{ext}}$ **do**
${\hat{Y}}_{\lambda_{2,k},\delta_{k}^{\text{int}},\delta_{k}^{\text{ext}};k}^{\kappa (i)}\leftarrow \mathrm{InverseLink}(X^{\kappa (i)}{\hat{\Gamma }}_{\cdot ,k}^{-\kappa (i)})$	$z_{k}\leftarrow \mathrm{PenaltyFactors}(w_{k}^{\text{int}},w_{k}^{\text{ext}},\delta_{k}^{\text{int}},\delta_{k}^{\text{ext}})$
**end for**	${\hat{\Gamma }}_{\cdot ,k}^{-\kappa (i)}\leftarrow \mathrm{AdCoLasso}(y^{\left(k),-\kappa (i\right)},X^{\left(k),-\kappa (i\right)},\lambda_{2,k},z_{k})$
**end for**	${\hat{y}}_{\lambda_{2,k},\delta_{k}^{\text{int}},\delta_{k}^{\text{ext}}}^{\left(k),\kappa (i\right)}\leftarrow \mathrm{InverseLink}(X^{\left(k),\kappa (i\right)}{\hat{\Gamma }}_{\cdot ,k}^{-\kappa (i)})$
**end for**	**end for**
**for** *k* in 1 to *q* **do**	**end for**
$\left\{\lambda_{2,k},\delta_{k}^{\text{int}},\delta_{k}^{\text{ext}}\}\leftarrow \mathrm{MinimiseMetric}(Y_{\cdot ,k},{\hat{Y}}_{\cdot ,\cdot ,\cdot ;k}\right)$	**end for**
**end for**	**for** *k* in 1 to *q* **do**
**Parameter estimation**	$\left\{\lambda_{2,k},\delta_{k}^{\text{int}},\delta_{k}^{\text{ext}}\}\leftarrow \mathrm{MinimiseMetric}(y^{\left(k\right)},{\hat{y}}_{\cdot ,\cdot ,\cdot }^{\left(k\right)}\right)$
**for** *k* in 1 to *q* **do**	**end for**
${\hat{B}}_{\cdot ,k}\leftarrow \mathrm{ElasticNet}(Y_{\cdot ,k},X,\lambda_{1,k})$	**Parameter estimation**
**end for**	**for** *k* in 1 to *q* **do**
**for** *k* in 1 to *q* **do**	${\hat{B}}_{\cdot ,k}\leftarrow \mathrm{ElasticNet}(y^{\left(k\right)},X^{\left(k\right)},\lambda_{1,k})$
$\left\{w_{k}^{\text{int}},w_{k}^{\text{ext}}\}\leftarrow \mathrm{Weights}(\hat{B},k\right)$	**end for**
$z_{k}\leftarrow \mathrm{PenaltyFactors}(w_{k}^{\text{int}},w_{k}^{\text{ext}},\delta_{k}^{\text{int}},\delta_{k}^{\text{ext}})$	**for** *k* in 1 to *q* **do**
**end for**	$\left\{w_{k}^{\text{int}},w_{k}^{\text{ext}}\}\leftarrow \mathrm{Weights}(\hat{B},k\right)$
**for** *k* in 1 to *q* **do**	$z_{k}\leftarrow \mathrm{PenaltyFactors}(w_{k}^{\text{int}},w_{k}^{\text{ext}},\delta_{k}^{\text{int}},\delta_{k}^{\text{ext}})$
${\hat{\Gamma }}_{\cdot ,k}\leftarrow \mathrm{AdCoLasso}(y_{\cdot ,k},X,\lambda_{2,k},z_{k})$	**end for**
**end for**	**for** *k* in 1 to *q* **do**
	${\hat{\Gamma }}_{\cdot ,k}\leftarrow \mathrm{AdCoLasso}(y_{\cdot ,k},X^{\left(k\right)},\lambda_{2,k},z_{k})$
	**end for**

a Standard subprocedures (details omitted): ElasticNetCV^*^ (optimising regularisation parameter), ElasticNet^*^ (estimating regression coefficients), InverseLink^*^ (transforming linear predictor to predicted value), MinimiseMetric^*^ (selecting optimal hyperparameters). Subprocedures described in Section Methods: *Weights* (calculating internal and external weights), *PenaltyFactors* (calculating penalty factors), AdCoLasso^*^ (fitting sign-aware adaptive lasso regression). The subprocedures marked with an asterisk differ between linear regression (numerical outcome) and logistic regression (binary outcome). The inclusion or exclusion of all samples from fold *i* is indicated by κ(i) and −κ(i), respectively.

## 3 Simulation

### 3.1 Data generating process

We repeatedly simulate data for multi-task learning and transfer learning to test the proposed method.

For multi-task learning, we repeatedly simulate q=3 targets and p=200 features for a small number of training samples (n=100) and a large number of testing samples (m=10,000), where the targets are indexed by k∈{1,…,q}, the features by j∈{1,…,p}, and the samples by i∈{1,…,n+m}. In each iteration, we simulate (i)  the rows of the (n+m)×q feature matrix X from a multivariate Gaussian distribution with a decreasing correlation structure (i.e. $X_{i,\cdot }∼N(0,R)$, where $R_{j,l}=\rho^{\left|j-l\right|}$ and ρ=0.5), (ii)  the p×q effect matrix B as shown below, and (iii)  the (n+m)×q target matrix Y=XB+E, where E is Gaussian noise ($E_{i,k}∼N(0,1)$).

For transfer learning, we repeatedly simulate q=3 datasets with one target and p=200 features for small numbers of training samples ($n_{1}=50$, $n_{2}=100$ and $n_{3}=200$) and large numbers of testing samples ($m_{1}=m_{2}=m_{3}=\text{10,000}$), where the datasets are indexed by k∈{1,…,q}, the features by j∈{1,…,p}, and the samples by $i\in \{1,\ldots ,n_{k}+m_{k}\}$. In each iteration, we simulate (i)  the rows of the $(n_{k}+m_{k})\times q$ feature matrices $X^{\left(k\right)}$ from multivariate Gaussian distributions with decreasing correlation structures (i.e. $X_{i,\cdot }^{\left(k\right)}∼N(0,R^{\left(k\right)})$, where $R_{j,l}^{\left(k\right)}=\rho_{k}^{\left|j-l\right|}$ and $\rho_{k}=0.5$), (ii)  the p×1 effect vectors $B_{\cdot ,k}$ as shown below, and (iii)  the $(n_{k}+m_{k})\times 1$ target vectors $y^{\left(k\right)}=X^{\left(k\right)}B_{\cdot ,k}+\epsilon^{\left(k\right)}$, where $\epsilon^{\left(k\right)}$ is Gaussian noise ($\epsilon_{i}^{\left(k\right)}∼\text{N}(0,1)$).

For multi-task learning and transfer learning, each problem obtains its own p×1 effect vector $B_{\cdot ,k}=\theta +\Delta_{\cdot ,k}$, where θ contains the effects shared by all problems and $\Delta_{\cdot ,k}$ contains the problem-specific effects. Given the probabilities of success for the shared effects ($\pi_{\theta }$) and the specific effects ($\pi_{\delta }$), we simulate the effects from mixture distributions of Bernoulli trials and standard Gaussian distributions (i.e. $\theta_{j}∼\text{Bern}(\pi_{\theta })\times N(0,1)$ and $\delta_{k,j}∼\text{Bern}(\pi_{\delta })\times N(0,1)$). If there are many shared and few specific effects (large $\pi_{\theta }$, small $\pi_{\delta }$), the problems are close to each other. But if there are few shared and many specific effects (small $\pi_{\theta }$, large $\pi_{\delta }$), the problems are far from each other. We consider all combinations of $\pi_{\theta }$ and $\pi_{\delta }$ in {0%,2.5%,5%}.

In short, we simulate related problems about the same features and the same samples (multi-task learning) or about the same features but different samples (transfer learning). While all problems share the same feature matrix in multi-task learning, each problem has its own feature matrix in transfer learning. Here, each prediction problem is supported by information from two other prediction problems. Table 2 shows the two data-generating processes.

**Table 2.** Data-generating processes for the simulation studies on multi-task learning (left) and transfer learning (right).

Multi-task learning	Transfer learning
*q* problems (targets), indexed by k∈{1,…,q}	*q* problems (datasets), indexed by k∈{1,…,q}
*p* features, indexed by j,l∈{1,…,p}	*p* features, indexed by j,l∈{1,…,p}
n+m samples, indexed by i∈{1,…,n+m}	$n_{k}+m_{k}$ samples, indexed by $i\in \{1,\ldots ,n_{k}+m_{k}\}$
**Features**	**Features**
(n+m)×p matrix X	$(n_{k}+m_{k})\times p$ matrices $X^{\left(k\right)}$
$X_{i,\cdot }∼N(0,R)$ , where $R_{j,l}=\rho^{\left|j-l\right|}$	$X_{i,\cdot }^{\left(k\right)}∼N(0,R^{\left(k\right)})$ , where $R_{j,l}^{\left(k\right)}=\rho_{k}^{\left|j-l\right|}$
**Effects**	**Effects**
p×q matrix B	p×q matrix B
$B_{\cdot ,k}=\theta +\Delta_{\cdot ,k}$	$B_{\cdot ,k}=\theta +\Delta_{\cdot ,k}$
$\theta_{j}∼\text{Bern}(\pi_{\theta })\times N(0,1)$	$\theta_{j}∼\text{Bern}(\pi_{\theta })\times N(0,1)$
$\Delta_{j,k}∼\text{Bern}(\pi_{\delta })\times N(0,1)$	$\Delta_{j,k}∼\text{Bern}(\pi_{\delta })\times N(0,1)$
**Targets**	**Targets**
(n+m)×q matrix Y	$(n_{k}+m_{k})\times 1$ vectors $y^{\left(k\right)}$
Y=XB+E	$y^{\left(k\right)}=X^{\left(k\right)}B_{\cdot ,k}+\epsilon^{\left(k\right)}$
$E_{i,k}∼N(0,1)$	$\epsilon_{i}^{\left(k\right)}∼\text{N}(0,1)$

### 3.2 Predictive and selection performance

**Figure 2.**
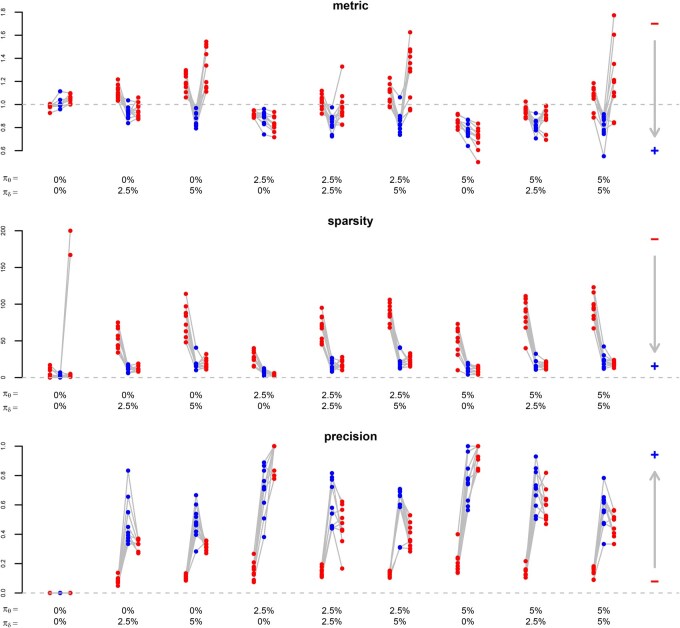
Multi-task learning. Comparison of different measures (rows) between the proposed method (blue points in the centre) and two available methods (Simon *et al.* 2013, R package glmnet: red points on the left; Chung and Keleş 2010, R package spls: red points on the right) in different simulation settings (columns), based on the average of three problems (tasks) for each repetition out of ten. Measures: performance metric (mean squared error on hold-out data, as a fraction of the one from standard lasso regression; a point below the dashed line means that multi-task learning improves predictions), sparsity (number of non-zero coefficients), precision (number of coefficients with correct signs divided by number of non-zero coefficients). The arrows point in the direction of improvement. Settings: percentage of features with a shared effect for all problems ($\pi_{\theta }$), percentage of features with a specific effect for each problem ($\pi_{\delta }$).

**Figure 3.**
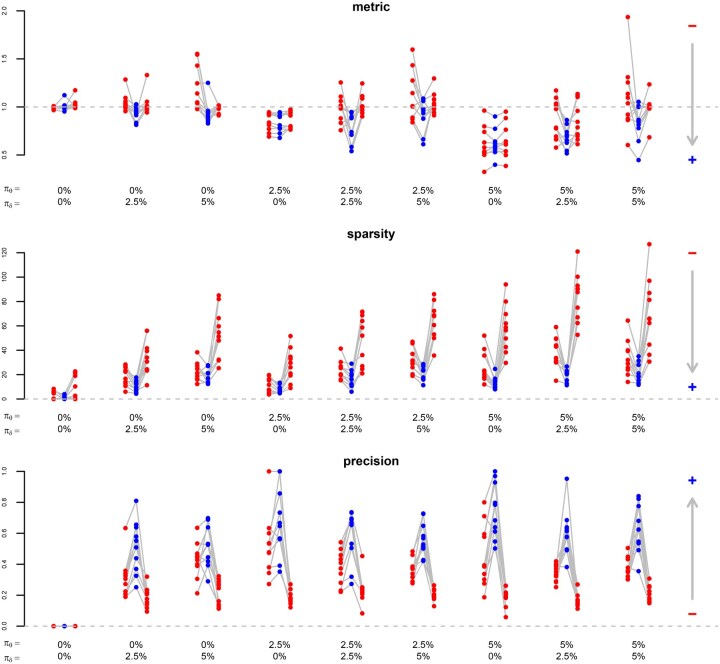
Transfer learning. Comparison of different measures (rows) between the proposed method (blue points in the centre) and two available methods (Tian and Feng 2023, R package glmtrans: red points on the left; Weaver and Lewinger 2019, R package xrnet: red points on the right) in different simulation settings (columns), based on the average of three problems (datasets) for each repetition out of ten. Measures: performance metric (mean squared error on hold-out data, as a fraction of the one from standard lasso regression; a point below the dashed line means that transfer learning improves predictions), sparsity (number of non-zero coefficients), precision (number of coefficients with correct signs divided by number of non-zero coefficients). The arrows point in the direction of improvement. Settings: percentage of features with a shared effect for all problems ($\pi_{\theta }$), percentage of features with a specific effect for each problem ($\pi_{\delta }$).

While we compare the proposed approach to multivariate Gaussian lasso regression from Simon *et al.* (2013, R package glmnet with family=“mgaussian”) and to sparse partial least squares (Chung and Keleş 2010, R package spls) in the case of multi-task learning, we compare it to transfer learning Gaussian lasso regression from Tian and Feng (2023, R package glmtrans) and to hierarchical regularised regression (Weaver and Lewinger 2019, R package xrnet) in the case of transfer learning. For sparse partial least squares, we specify the candidate values {3,…,10} for the hidden components and {0,0.1,…,0.9} for the thresholding parameter, and for hierarchical regularised regression, we provide the coefficients from lasso-like elastic net regression (α=0.95) as external data. Figures 2 and 3 show the results for multi-task learning and transfer learning, respectively.

Concerning the predictive performance, we make similar observations for multi-task learning and transfer learning. We choose the mean squared error as a performance metric, calculated on the testing data (hold-out method). The mean squared error decreases as the predictive performance improves. If there are neither shared effects ($\pi_{\theta }=0\%$) nor specific effects ($\pi_{\delta }=0\%$), the proposed and the available methods have a similar predictive performance as standard lasso regression. (Note that the proposed method reduces to the standard lasso if $\delta_{k}^{\text{int}}=\delta_{k}^{\text{ext}}=0$.) If there are some shared effects ($\pi_{\theta }=2.5\%$) but no specific effects ($\pi_{\delta }=0\%$), the proposed and the available methods outperform standard lasso regression, meaning that sharing information between problems is beneficial. This benefit is greater if there are many shared effects ($\pi_{\theta }=5\%$), which makes sense as multi-task learning and transfer learning should be more beneficial for closely related problems than for loosely related problems. If there are no shared effects ($\pi_{\theta }=0\%$) but specific effects ($\pi_{\delta }=2.5\%$ or $\pi_{\delta }=5\%$), the proposed method is more predictive than the available methods. It seems that adaptive penalisation, even without sharing information between problems, improves the predictive performance in these sparse simulation settings. (Note that the proposed method reduces to the adaptive lasso with weights obtained from the same dataset if $\delta_{k}^{\text{ext}}=0$.) Given a proportion of shared effects ($\pi_{\theta }=0\%$, $\pi_{\theta }=2.5\%$ or $\pi_{\theta }=5\%$), the performance of the proposed method relative to the available methods increases with the proportion of specific effects ($\pi_{\delta }=0\%$, $\pi_{\delta }=2.5\%$, or $\pi_{\delta }=5\%$). If there are many specific effects ($\pi_{\delta }=5\%$), only the proposed method shows a benefit with respect to the standard lasso.

Concerning the selection performance, we make different observations for multi-task learning and transfer learning. We assess two aspects of the selection performance, namely the sparsity (measured by the number of non-zero coefficients) and the precision (measured by the number of coefficients with correct signs divided by the number of non-zero coefficients). As the selection performance improves, the number of non-zero coefficients decreases and the proportion of correct signs increases. For multi-task learning, except if there are neither shared nor specific effects ($\pi_{\theta }=0\%$ and $\pi_{\delta }=0\%$), the proposed method and the method from Chung and Keleş (2010) estimate sparser and more precise models than the method from Simon *et al.* (2013). A possible explanation is that the multivariate method from Simon *et al.* (2013) by construction selects the same features for all tasks. For transfer learning, the proposed method estimates sparser and more precise models than the available methods if there are specific effects ($\pi_{\theta }=2.5\%$ or $\pi_{\theta }=5\%$). A possible explanation is that the transfer learning method from Tian and Feng (2023) first selects a shared set of features for all datasets and then adds a specific set of features for each dataset, and that the method from Weaver and Lewinger (2019) shrinks the coefficients towards a linear combination of the prior effects.

### 3.3 Computation time

Sharing information between problems increases the computation time. In the multi-task learning simulation, the proposed method is almost twice as slow as the slower one of the two available methods (computation time relative to standard lasso: 28.6; Simon *et al.* 2013, R package glmnet: 1.4; Chung and Keleş 2010, R package spls: 16.6). And in the transfer learning simulation, the proposed method is as slow as the slower one of the two available methods (computation time relative to standard lasso: 22.3; Tian and Feng 2023, R package glmtrans: 21.9; Weaver and Lewinger 2019, R package xrnet: 5.7). The relative computation times depend on the number of problems, number of features, and sample sizes.

### 3.4 Impact of sample size

**Figure 4.**
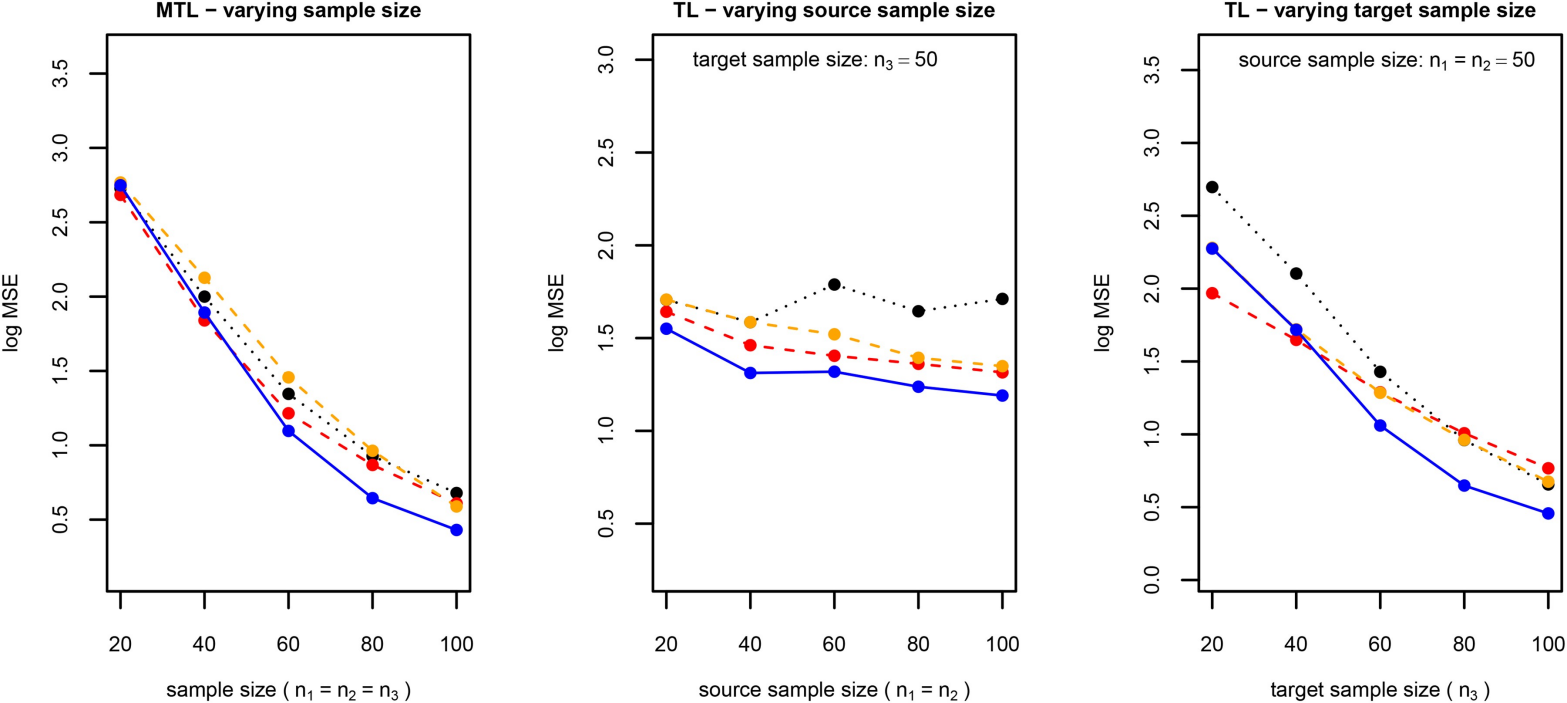
Sample size. Logarithmic transformation of mean squared error calculated on hold-out data (*y*-axis) against source or target sample size (*x*-axis), for the standard lasso (dotted black line), available methods (dashed red and orange lines), and the proposed method (solid blue line), based on 10 simulated datasets for each sample size. The available methods are those from Simon *et al.* (2013, R package glmnet, red) and Chung and Keleş (2010, R package spls, orange) for multi-task learning (mtl, left) and those from Tian and Feng (2023, R package glmtrans, red) and Weaver and Lewinger (2019, R package xrnet, orange) for transfer learning (tl, centre and right). While the mean squared error is averaged across all three targets in the case of multi-task learning, it is only reported for the target dataset (k=3) in the case of transfer learning.

Fixing the proportion of shared effects at $\pi_{\theta }=5\%$ and the one of specific effects at $\pi_{\theta }=2.5\%$, we repeat the simulation study under different sample sizes. Figure 4 shows how the out-of-sample predictive performance depends on the sample size. In the case of transfer learning, we distinguish between the sample sizes of the source datasets (i.e. the supporting datasets) and the target dataset (i.e. the dataset of interest). The proposed method is more predictive than the available methods, with the following exceptions: (i)  In the case of multi-task learning under a small sample size, the method from Simon *et al.* (2013, R package glmnet) is more predictive. This method selects a common set of features for all tasks. (ii)  In the case of transfer learning under a small target sample size, the methods from Tian and Feng (2023, R package glmtrans) and Weaver and Lewinger (2019, R package xrnet) are more predictive. The former method first estimates a common model for all datasets and then the deviations for the target dataset, and the latter method penalises the differences between the models for the source dataset and the one for the target dataset. We therefore conclude that these available methods are more suitable for small sample sizes (multi-task learning) or small target sample sizes (transfer learning). By contrast, the proposed method needs a sufficiently large (target) sample size for model training.

## 4 Application

### 4.1 Data preparation

We searched for datasets on treatment response in three autoimmune diseases, namely inflammatory bowel disease (ibd), rheumatoid arthritis (ra), and multiple sclerosis (ms), on a public resource of uniformly processed rna-seq data (Wilks *et al.* 2021, R package recount3). Restricting the search to prospective datasets with at least 40 samples, we found four datasets on treatment response in ibd (Tew *et al.* 2016, Haberman *et al.* 2019, Verstockt *et al.* 2019, 2020). We also included four related datasets, namely a cross-sectional dataset on disease activity in ibd (Boyd *et al.* 2018), a prospective dataset on drug-free remission in ra (Baker *et al.* 2019), a prospective dataset on treatment response in juvenile idiopathic arthritis (Moncrieffe *et al.* 2017), and a cross-sectional dataset on disease activity in ra (Goldberg *et al.* 2018). Table 3 includes some details on the five datasets related to treatment response in ibd and the three datasets related to treatment response in ra. The aim is to apply transfer learning to these datasets, separately for ibd and ra. This means that each classification problem is supported by information from four or two other classification problems, respectively.

**Table 3.** Datasets related to treatment response in ibd (1–5) and to treatment response in ra (6–8).

Tew *et al.* (2016, srp063496)	70 uc patients were treated with etrolizumab. Gene expression was measured in colonic biopsies at baseline. During follow-up at week 10, 12 remissions and 58 non-remissions were observed.
Haberman *et al.* (2019, srp129004)	206 new-onset pediatric uc patients were treated with mesalamine or corticosteroids. Gene expression was measured in rectal mucosal biopsies at baseline. During follow-up at week 4, 105 remissions and 101 non-remissions were observed.
Verstockt *et al.* (2019, erp113396)	43 ibd patients were treated with two different tumour necrosis factor inhibitors, namely 27 with adalimumab and 16 with infliximab. Mucosal gene expression was measured in inflamed biopsies. Treatment response was endoscopic remission during follow-up, with 18 responders and 25 non-responders.
Verstockt *et al.* (2020, erp114636)	47 ibd patients, namely 20 with Crohn’s disease (cd) and 27 with ulcerative colitis (uc), were treated with vedolizumab. Gene expression was measured in biopsies from inflamed colon. Treatment response is endoscopic remission during follow-up, with 24 responders and 23 non-responders.
Boyd *et al.* (2018, srp100787)^a^	64 ibd patients, namely 22 with cd and 42 with uc, were assessed for disease activity. Gene expression was measured in pinch biopsies from the descending colon at baseline. Disease activity was defined based on two different scores for cd and uc, leading to 20 patients with an inactive and 44 patients with an active disease at baseline.
Baker *et al.* (2019, srp169062)^b^	44 patients with ra were observed for drug-free remission. Gene expression was measured in CD4+ T cells from peripheral blood samples. Drug-free remission was defined based on a disease activity score in multiple joints, leading to 21 patients without flares and 23 patients with flares.
Moncrieffe *et al.* (2017, srp074736)^b^	46 patients with juvenile idiopathic arthritis, which is different from ra, were treated with methotrexate. Gene expression was measured in peripheral blood mononuclear cells. Treatment response was defined based on six scores, with a large increase in at least three scores and a large decrease in at most one score, leading to 29 responders and 17 non-responders.
Goldberg *et al.* (2018, srp155483)^a^	48 patients with ra were observed. Gene expression was measured in white blood cells from peripheral blood. 24 patients under remission are to be compared to 3+15+6=24 patients with low, moderate or high disease activity.

a No prospective dataset on treatment response but a cross-sectional dataset on disease activity.

b No dataset on treatment response in ibd or ra but closely related.

Restricting the analysis to the 22,321 protein-coding genes from the 63,856 transcripts, we select the 5,000 most variably expressed protein-coding genes across all datasets. Specifically, in each dataset, we rank the genes in ascending order of their variances, take the mean of these ranks across all datasets, and retain the genes with the top 5,000 highest mean ranks. For each dataset, we calculate the library sizes by summing the expression values across all transcripts (not only the selected protein-coding genes) for each sample, normalise the samples for different library sizes and compositional biases using the trimmed mean of M-values method (Robinson and Oshlack 2010, R package edgeR), stabilise the variance with the Anscombe transform ($x\to 2\sqrt{x+3/8}$), and standardise the features (zero mean, unit variance).

### 4.2 Data exploration

To explore the relationships between the different datasets, we trained logistic ridge regression on each dataset. We use ridge rather than lasso regularisation to facilitate comparisons across datasets, because it leads to non-zero coefficients for all features. Note that correlated ridge regression coefficients (dense models) are neither necessary nor sufficient for successful transfer learning in lasso regression (sparse models).

First, we compared the regression coefficients from the 8 datasets with Spearman’s correlation test, maintaining the family-wise error rate for the 82=28 pairwise combinations at 5% with the Bonferroni correction. We observe that the regression coefficients from the 5 ibd datasets are significantly correlated (*p*-value ≤0.05/28 for all 10 pairwise combinations). While the correlation is significantly *positive* for all 6 pairwise combinations not including the dataset from Verstockt *et al.* (2020), it is significantly *negative* for all 4 pairwise combinations including the dataset from Verstockt *et al.* (2020). We therefore inverted the target from Verstockt *et al.* (2020), by exchanging the class labels for responders and non-responders. Table 4 shows the correlation coefficients between the regression coefficients from the different datasets, after this inversion. The regression coefficients are significantly positively correlated in 10 out of 10 comparisons between ibd datasets, 1 out of 3 comparisons between ra datasets, and 6 out of 15 comparisons between ibd and ra datasets (*p*-value ≤0.05/28). It is therefore more promising to share information among the ibd datasets than among the ra datasets or between the ibd and the ra datasets.

**Table 4.** Spearman correlation coefficients between the ridge regression coefficients from different datasets. Pairwise combinations of datasets with significantly correlated regression coefficients are highlighted, with black colour for nominal significance (*p*-value ≤.05) and stars for adjusted significance (*p*-value ≤.05/28). We expect a correlation coefficient close to 0 for unrelated problems and close to 1 for identical problems.

	Tew *et al.* (2016)	Haberman *et al.* (2019)	Verstockt *et al.* (2019)	Verstockt *et al.* (2020)	Boyd *et al.* (2018)	Baker *et al.* (2019)	Moncrieffe *et al.* (2017)	Goldberg *et al.* (2018)
Tew *et al.* (2016)	–	0.23${\;}^{⋆}$	0.34${\;}^{⋆}$	0.22${\;}^{⋆}$	0.18${\;}^{⋆}$	−0.02	−0.01	−0.01
Haberman *et al.* (2019)	0.23${\;}^{⋆}$	–	0.26${\;}^{⋆}$	0.29${\;}^{⋆}$	0.22${\;}^{⋆}$	0.08${\;}^{⋆}$	0.06${\;}^{⋆}$	−0.01
Verstockt *et al.* (2019)	0.34${\;}^{⋆}$	0.26${\;}^{⋆}$	–	0.35${\;}^{⋆}$	0.15${\;}^{⋆}$	−0.02	0.08${\;}^{⋆}$	0.04
Verstockt *et al.* (2020)	0.22${\;}^{⋆}$	0.29${\;}^{⋆}$	0.35${\;}^{⋆}$	–	0.15${\;}^{⋆}$	0.05${\;}^{⋆}$	0.07${\;}^{⋆}$	−0.04
Boyd *et al.* (2018)	0.18${\;}^{⋆}$	0.22${\;}^{⋆}$	0.15${\;}^{⋆}$	0.15${\;}^{⋆}$	–	0.10${\;}^{⋆}$	−0.04	−0.04
Baker *et al.* (2019)	−0.02	0.08${\;}^{⋆}$	−0.02	0.05${\;}^{⋆}$	0.10${\;}^{⋆}$	–	0.02	0.02
Moncrieffe *et al.* (2017)	−0.01	0.06${\;}^{⋆}$	0.08${\;}^{⋆}$	0.07${\;}^{⋆}$	−0.04	0.02	–	−0.05${\;}^{⋆}$
Goldberg *et al.* (2018)	−0.01	−0.01	0.04	−0.04	−0.04	0.02	−0.05${\;}^{⋆}$	–

Second, we assessed the cross-dataset predictive performance, i.e. by training the logistic regression on a dataset and testing it on the other datasets. Based on the out-of-sample predicted probabilities, we calculated the area under the receiver operating characteristic curve (roc-auc) for each pairwise permutation of datasets. To examine whether a classifier significantly outperforms a random classifier, we used the one-sided Mann-Whitney *U* test to test whether the ranks of the predicted probabilities of treatment response are significantly higher for the responders than for the non-responders. Table 5 shows the roc-auc for each pairwise permutation of datasets (off-diagonal entries). While models trained on an ibd dataset and tested on another ibd dataset tend to significantly outperform a random classifier at the nominal 5% level (*p*-value ≤0.05 for 16 out of 20 pairwise permutations), this is rarely the case for models that are trained or tested on an ra dataset (*p*-value ≤0.05 for 2 out of 36 pairwise permutations). This suggests that transfer learning might be beneficial for the ibd datasets.

**Table 5.** Out-of-sample area under the receiver operating characteristic curve (roc-auc) from logistic ridge regression trained on the dataset in the row and tested on the dataset in the column (off-diagonal entries), or cross-validated roc-auc from logistic lasso regression trained and tested on the same dataset by 10-fold external cross-validation (diagonal entries, between brackets). The roc-auc of a random classifier is 0.5, while that of a perfect classifier is 1.0. Entries on and off the diagonal are not comparable. Predictions that are significantly better than random predictions (according to the one-sided Mann-Whitney *U* test for testing whether the ranks of the predicted probabilities are significantly higher for the cases than for the controls) are highlighted, with black colour for nominal significance (*p*-value ≤.05) and stars for adjusted significance (*p*-value ≤.05/64).

	Tew *et al.* (2016)	Haberman *et al.* (2019)	Verstockt *et al.* (2019)	Verstockt *et al.* (2020)	Boyd *et al.* (2018)	Baker *et al.* (2019)	Moncrieffe *et al.* (2017)	Goldberg *et al.* (2018)
Tew *et al.* (2016)	(0.69)	0.61	0.71	0.66	0.78${\;}^{⋆}$	0.48	0.51	0.43
Haberman *et al.* (2019)	0.55	(0.54)	0.66	0.72	0.79${\;}^{⋆}$	0.58	0.56	0.40
Verstockt *et al.* (2019)	0.62	0.59	(0.44)	0.72	0.72	0.44	0.52	0.56
Verstockt *et al.* (2020)	0.57	0.59	0.65	(0.49)	0.74	0.53	0.57	0.48
Boyd *et al.* (2018)	0.58	0.65${\;}^{⋆}$	0.67	0.67	(0.87)${\;}^{⋆}$	0.65	0.49	0.46
Baker *et al.* (2019)	0.48	0.54	0.46	0.53	0.76${\;}^{⋆}$	(0.46)	0.50	0.51
Moncrieffe *et al.* (2017)	0.44	0.50	0.59	0.62	0.42	0.52	(0.44)	0.45
Goldberg *et al.* (2018)	0.48	0.52	0.58	0.44	0.41	0.46	0.46	(0.54)

In addition, we trained and tested logistic lasso regression on each dataset using 10-fold external cross-validation. Table 5 shows the cross-validated roc-auc for each dataset (diagonal entries). For the datasets from Tew *et al.* (2016) and Boyd *et al.* (2018), the predictions are significantly better than those from a random classifier at the nominal 5% level (*p*-value ≤0.05). For the other datasets, standard lasso regression fails to capture any predictive signal.

### 4.3 Transfer learning

We compared the proposed method to standard lasso regression, the transfer learning method from Tian and Feng (2023, R package glmtrans), and the transfer learning method from Weaver and Lewinger (2019, R package xrnet), in the application on ibd and the application on ra. To estimate the predictive performance of different methods, we used five times 10-fold external cross-validation. This involves splitting each dataset 5 times into 10 external folds, and using 9 external folds for training and 1 external fold for testing in each repetition. To assess the model sparsity of different methods, we also refit each model 5 times on each full dataset, using different internal folds in each repetition. The repetitions introduce variability in hyperparameter optimisation due to different splits of the samples into folds. For each dataset and each model, we thereby obtained five times a cross-validated roc-auc and five times a number of non-zero coefficients.

**Figure 5.**
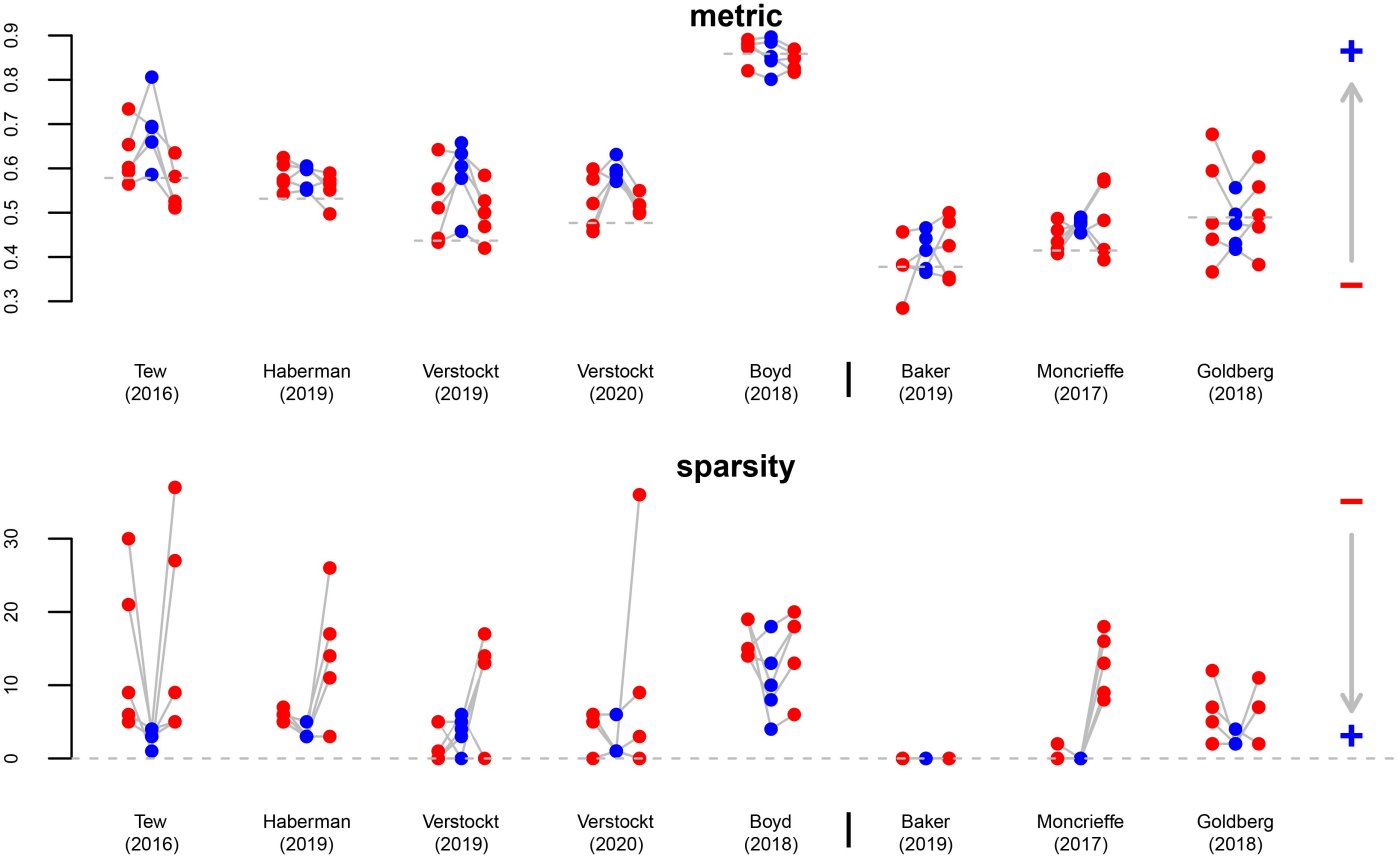
Comparison of different measures (rows) between the proposed method (blue points in the centre) and two available methods (Tian and Feng 2023, R package glmtrans: red points on the left; Weaver and Lewinger 2019, R package xrnet: red points on the right) in different applications (blocks of columns), based on 5 repetitions of 10-fold cross-validation. Measures: performance metric (cross-validated auc, the dashed lines indicate the mean from standard lasso regression), sparsity (number of non-zero coefficients). The arrows point in the direction of improvement. Applications: datasets related to treatment response in ibd (left), datasets related to treatment response in ra (right).

Figure 5 shows the performance of the proposed and the available transfer learning methods. For the ibd application, the proposed method reaches not only a higher mean cross-validated roc-auc (across 5 datasets and 5 repetitions) than the available methods (glmtrans: 0.62, sparselink: 0.66, xrnet: 0.59) but also a lower mean number of non-zero coefficients (glmtrans: 8.56, sparselink: 4.52, xrnet: 13.36). For the ra application, none of the three transfer learning methods reaches a mean roc-auc (across 3 datasets and 5 repetitions) above 0.50 (i.e. the performance of a random classifier), meaning that transfer learning cannot compensate for the weak predictive signal in the individual datasets.

In the ibd application, the proposed method increases the mean roc-auc (across five datasets and five repetitions) of logistic lasso regression from 0.58 to 0.66, with important differences between datasets. The increase in mean roc-auc (across five repetitions) is largest for the three prospective datasets on treatment response with less than 25 samples in the minority class, namely from 0.58 to 0.69 for Tew *et al.* (2016), from 0.44 to 0.59 for Verstockt *et al.* (2019), and from 0.48 to 0.60 for Verstockt *et al.* (2020). The proposed method only slightly increases the mean roc-auc from 0.53 to 0.58 for the prospective dataset on treatment response with more than 100 samples in the minority class (Haberman *et al.* 2019) and leaves it unchanged at 0.86 for the cross-sectional dataset on disease activity (Boyd *et al.* 2018). Information from problems with a strong signal appears to be useful for addressing problems with a weak signal, but not vice versa.

**Figure 6.**
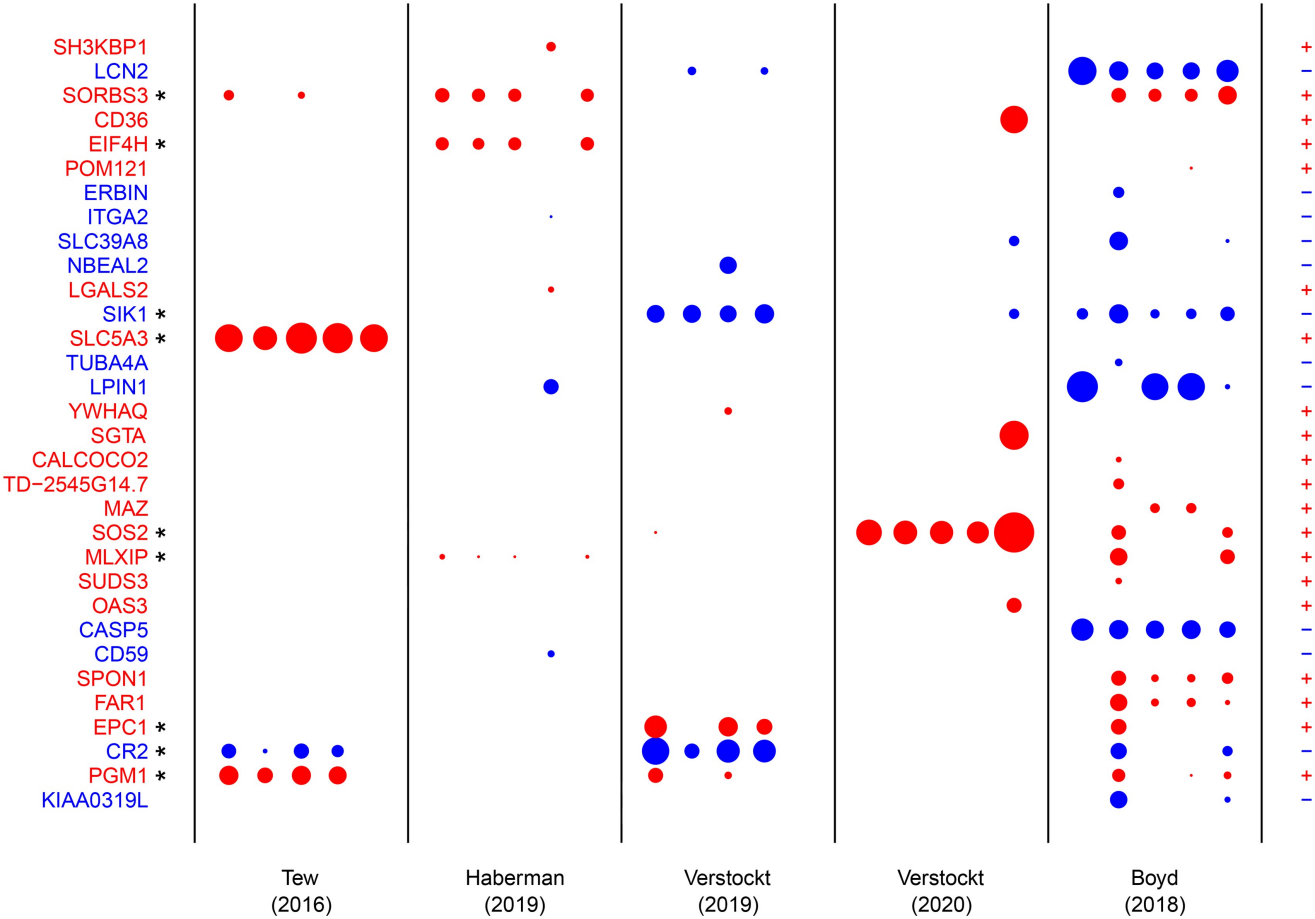
Standardised coefficients from the proposed transfer learning method for the datasets related to treatment response in ibd, based on five repetitions with different internal folds. Only genes selected for at least one dataset are shown. Blue (red) colour indicates that a gene is estimated to decrease (increase) the probability of treatment response (datasets 1–4) or disease inactivity (dataset 5), with the area of the circles proportional to the absolute value of the estimated effects. An asterisk indicates that a gene is selected in at least three out of five repetitions for at least one prospective dataset on treatment response (datasets 1–4). A plus (minus) sign indicates that a gene receives non-negative (non-positive) mean coefficients for all datasets.

Figure 6 shows the coefficients of the selected genes for the ibd datasets. Genes that are selected in at least 3 out of 5 repetitions in any of the prospective dataset on treatment response (excluding the cross-sectional dataset on disease activity) are *SORBS3*, *EIF4H*, *SLC5A3*, *SOS2*, *MLXIP*, *EPC1*, and *PGM1* with positive coefficients, and *SIK1* and *CR2* with negative coefficients. For none of these genes, however, we could find evidence in the scientific literature of direct effects on ibd treatment response.

## 5 Discussion

We designed the proposed method to estimate sparse but predictive models for each prediction or classification problem. Our adaptive penalisation scheme encourages the method to select the same features as well as to estimate the same signs and the same sizes for their effects in all problems. As we do not impose any strict constraints on the models, however, the method might select different features as well as estimate different signs and different sizes for their effects.

This low degree of harmonisation is only one possible compromise between estimating a common model for all problems and estimating a separate model for each problem. A higher degree of harmonisation might involve a common set of non-zero coefficients, a common set of non-positivity and non-negativity constraints on the coefficients for all problems, or penalised differences in coefficients between problems. This might not only be beneficial for interpretation in some applications but also for predictivity under low signal-to-noise ratios (i.e. due to small sample sizes or many weak effects).

While all problems might be of interest in some applications, only some problems might be of interest in other applications. We can then distinguish between problems providing prior information (i.e. source datasets in transfer learning or secondary outcomes in multi-task learning) and problems receiving prior information (i.e. target datasets in transfer learning or primary outcomes in multi-task learning). We expect that sharing information between problems is useful when the supporting problems are relatively straightforward and the supported problems are relatively difficult (e.g. due to small sample sizes or outlier contamination).

The proposed approach involves two hyperparameters for determining the influence of the internal and external weights in each problem. If there are three (or more) problems, however, two (or more) problems influence the external weights. Instead of giving equal weight to each external problem, it might be advantageous to give more weight to informative ones and less weight to uninformative ones. A computationally expensive solution would be to tune one weight for each combination of models. Alternatively, it might be possible to construct weights based on the predictivity of the initial coefficients from each problem for the targets from the other problems.

Federated learning allows researchers to build models based on multiple datasets without sharing the datasets. The proposed method could be implemented for federated transfer learning settings, because each first-stage regression and each second-stage regression only exploits a single dataset while information is transferred between datasets by means of the initial coefficients from the first-stage regressions. This is suitable for a decentralised federated setting, where each node first trains its local model based on its dataset, shares this model with the other nodes, and finally retrains its local model. This procedure shares information between datasets, not to obtain a global model for all datasets together but to improve the local model for each dataset separately.

## Data Availability

The R package sparselink is available on GitHub (https://github.com/rauschenberger/sparselink) and cran (https://cran.r-project.org/package=sparselink) under the mit license and includes the analysis code in a vignette. Data are accessible via the R package recount3 (Wilks *et al.* 2021), available on Bioconductor (https://bioconductor.org/packages/recount3/), under the project identifiers (insdc accession numbers) srp063496 (Tew *et al.* 2016), srp129004 (Haberman *et al.* 2019), erp113396 (Verstockt *et al.* 2019), erp114636 (Verstockt *et al.* 2020), srp100787 (Boyd *et al.* 2018), srp074736 (Moncrieffe *et al.* 2017), srp169062 (Baker *et al.* 2019), and srp155483 (Goldberg *et al.* 2018).
